# Supplementary material for: Demographic compensation occurs in populations of Quercus oleoides Schltdl & Cham in fragments across an altitudinal gradient
Source: PeerJ. 2025 Feb 24;13:e18980. doi: 10.7717/peerj.18980 (PMC11867044; doi:10.7717/peerj.18980)
Supplement: Supplemental Information 1 [file peerj-13-18980-s001.docx]

Appendix A

|  | | | | | | |
| --- | --- | --- | --- | --- | --- | --- |
| Table 1. Transition matrices and population growth rates (λ) by period of *Quercus oleoides* population of Miramar, Veracruz center state, Mexico. d diameter at breast height (cm), h height (h). | | | | | | |
| Cycle 2016-17  λ=1.121, λ stabilizes in 12 years. | | | | | | |
| Size | Seeds | <1.3 h | >0-10 d | >10-20 d | >20-30 d | >30 d |
| Seeds | 0 | 0 | 571.5 | 5000 | 10000 | 9000 |
| <1.3 | 0.00008 | 0.1314 | 0 | 0 | 0 | 0 |
| >0-10 | 0 | 0.7439 | 0.74571 | 0 | 0 | 0 |
| >10-20 | 0 | 0 | 0.1143 | 0.6727 | 0 | 0 |
| >20-30 | 0 | 0 | 0 | 0.2727 | 0.8966 | 0 |
| >30 | 0 | 0 | 0 | 0 | 0.1034 | 0.9828 |
| Cycle 2017-18  λ=1.1099, λ stabilizes in 9 years. | | | | | | |
| Seeds | 0 | 0 | 157.9 | 1000 | 6000 | 6500 |
| <1.3 | 0.00006 | 0.2063 | 0 | 0 | 0 | 0 |
| >0-10 | 0 | 0.7619 | 0.7632 | 0 | 0 | 0 |
| >10-20 | 0 | 0 | 0.1579 | 0.6 | 0 | 0 |
| >20-30 | 0 | 0 | 0 | 0.4 | 0.6829 | 0 |
| >30 | 0 | 0 | 0 | 0 | 0.2927 | 0.982 |
| Cycle 2018-2019  λ=1.009, λ stabilizes in 24 years | | | | | | |
| Seeds | 0 | 0 | 156.6 | 3000 | 8000 | 8000 |
| <1.3 | 0.000011 | 0.029 | 0 | 0 | 0 | 0 |
| >0-10 | 0 | 0.8235 | 0.7836 | 0 | 0 | 0 |
| <10-20 | 0 | 0 | 0.0522 | 0.9333 | 0 | 0 |
| <20-30 | 0 | 0 | 0 | 0.0666 | 0.9783 | 0 |
| >30 | 0 | 0 | 0 | 0 | 0.0217 | 0.9828 |

| Table 2. Transition matrices and population growth rates (λ) by period of *Quercus oleoides* population of Mesa de Veinticuatro, Veracruz center state, Mexico. d diameter at breast height (cm), h height (h). | | | | | | |
| --- | --- | --- | --- | --- | --- | --- |
| Cycle 2016-17  λ=1.014, λ stabilizes in 16 years. | | | | | | |
| Size | Seeds | <1.3 h | >0-10 d | >10-20 d | >20-30 d | >30 d |
| Seeds | 0 | 0 | 2552.6 | 3000 | 8000 | 9000 |
| <1.3 | 0.000006 | 0.0313 | 0 | 0 | 0 | 0 |
| >0-10 | 0 | 0.625 | 0.2632 | 0 | 0 | 0 |
| >10-20 | 0 | 0 | 0.6842 | 0.6234 | 0 | 0 |
| >20-30 | 0 | 0 | 0 | 0.3389 | 0.772 | 0 |
| >30 | 0 | 0 | 0 | 0 | 0.2056 | 0.988 |
| Cycle 2017-18  λ=0.996, λ stabilizes in 25 years. | | | | | | |
| Seeds | 0 | 0 | 36 | 900 | 3000 | 3000 |
| <1.3 | 0.000009 | 0.0667 | 0 | 0 | 0 | 0 |
| >0-10 | 0 | 0.8 | 0.88 | 0 | 0 | 0 |
| >10-20 | 0 | 0 | 0.04 | 0.5 | 0 | 0 |
| >20-30 | 0 | 0 | 0 | 0.4938 | 0.7182 | 0 |
| >30 | 0 | 0 | 0 | 0 | 0.2818 | 0.988 |
| Cycle 2018-2019  λ=1.009, λ stabilizes in 14 years. | | | | | | |
| Seeds | 0 | 0 | 235 | 2000 | 4000 | 4000 |
| <1.3 | 0.00002 | 0.1111 | 0 | 0 | 0 | 0 |
| >0-10 | 0 | 0.7778 | 0.6176 | 0 | 0 | 0 |
| <10-20 | 0 | 0 | 0.1176 | 0.8205 | 0 | 0 |
| <20-30 | 0 | 0 | 0 | 0.1667 | 0.958 | 0 |
| >30 | 0 | 0 | 0 | 0 | 0.0336 | 0.988 |

| Table 3. Transition matrices and population growth rates (λ) by period of *Quercus oleoides* population of Otates, Veracruz center state, Mexico. d diameter at breast height (cm), h height (h). | | | | | | |
| --- | --- | --- | --- | --- | --- | --- |
| Cycle 2016-17  λ=1.024, λ stabilizes in 10 years. | | | | | | |
| Size | Seeds | <1.3 h | >0-10 d | >10-20 d | >20-30 d | >30 d |
| Seeds | 0 | 0 | 1429.7 | 8500 | 9000 | 5000 |
| <1.3 | 0.00002 | 0.6689 | 0 | 0 | 0 | 0 |
| >0-10 | 0 | 0.1689 | 0.785 | 0 | 0 | 0 |
| >10-20 | 0 | 0 | 0.1682 | 0.9256 | 0 | 0 |
| >20-30 | 0 | 0 | 0 | 0.0556 | 0.8182 | 0 |
| >30 | 0 | 0 | 0 | 0 | 0.0909 | 0.9861 |
| Cycle 2017-18  λ=1.018, λ stabilizes in 13 years. | | | | | | |
| Seeds | 0 | 0 | 444 | 3000 | 3000 | 1000 |
| <1.3 | 0.00004 | 0.3017 | 0 | 0 | 0 | 0 |
| >0-10 | 0 | 0.6293 | 0.8148 | 0 | 0 | 0 |
| >10-20 | 0 | 0 | 0.1481 | 0.9322 | 0 | 0 |
| >20-30 | 0 | 0 | 0 | 0.0254 | 0.0375 | 0 |
| >30 | 0 | 0 | 0 | 0 | 0.0625 | 0.986 |
| Cycle 2018-2019  λ=1.031, λ stabilizes in 19 years. | | | | | | |
| Seeds | 0 | 0 | 360 | 2000 | 2000 | 1500 |
| <1.3 | 0.00007 | 0.01961 | 0 | 0 | 0 | 0 |
| >0-10 | 0 | 0.8235 | 0.6832 | 0 | 0 | 0 |
| <10-20 | 0 | 0 | 0.1801 | 0.9603 | 0 | 0 |
| <20-30 | 0 | 0 | 0 | 0.0079 | 0.9444 | 0 |
| >30 | 0 | 0 | 0 | 0 | 0.0556 | 0.9861 |

| Table 4. Average vector (population structure) of *Quercus oleoides* populations, Veracruz center state, Mexico. S$\bar{X}$ mean standard deviation. | | | | | | |
| --- | --- | --- | --- | --- | --- | --- |
|  | Miramar | | Mesa de Veinticuatro | | Otates | |
|  | Mean | S$\bar{X}$ | Mean | S$\bar{X}$ | Mean | S$\bar{X}$ |
| Seeds | 19128 | 3153.5 | 13230 | 4757.7 | 12412 | 5784.2 |
| <1.3 | 39.333 | 6.658 | 141.000 | 2.000 | 46.333 | 3.055 |
| >0-10 | 76.000 | 7.810 | 94.667 | 1.528 | 102.667 | 1.528 |
| <10-20 | 32.000 | 2.000 | 130.667 | 2.517 | 46.667 | 2.887 |
| <20-30 | 64.333 | 5.132 | 68.000 | 1.000 | 100.000 | 3.000 |
| >30 | 57.333 | 4.041 | 84.000 | 1.000 | 70.667 | 2.309 |

| Table 5. Elasticity matrices by period of *Quercus oleoides* population of Miramar, Veracruz center state, Mexico. d diameter at breast height (cm), h height (h). | | | | | | |
| --- | --- | --- | --- | --- | --- | --- |
| Cycle 2016-17 | | | | | | |
| Size | Seeds | <1.3 h | >0-10 d | >10-20 d | >20-30 d | >30 d |
| Seeds | 0.000 | 0.000 | 0.006 | 0.013 | 0.033 | 0.022 |
| <1.3 | 0.0741732 | 0.010 | 0.000 | 0.000 | 0.000 | 0.000 |
| >0-10 | 0.000 | 0.0741732 | 0.147 | 0.000 | 0.000 | 0.000 |
| >10-20 | 0.000 | 0.000 | 0.0681253 | 0.102 | 0.000 | 0.000 |
| >20-30 | 0.000 | 0.000 | 0.000 | 0.0546473 | 0.218 | 0.000 |
| >30 | 0.000 | 0.000 | 0.000 | 0.000 | 0.022 | 0.156 |
| Cycle 2017-18 | | | | | | |
| Seeds | 0.000 | 0.000 | 0.001 | 0.003 | 0.015 | 0.041 |
| <1.3 | 0.0605728 | 0.014 | 0.000 | 0.000 | 0.000 | 0.000 |
| >0-10 | 0.000 | 0.0605728 | 0.138 | 0.000 | 0.000 | 0.000 |
| >10-20 | 0.000 | 0.000 | 0.0592481 | 0.071 | 0.000 | 0.000 |
| >20-30 | 0.000 | 0.000 | 0.000 | 0.05659512 | 0.093 | 0.000 |
| >30 | 0.000 | 0.000 | 0.000 | 0.000 | 0.041 | 0.346 |
| Cycle 2018-2019 | | | | | | |
| Seeds | 0.000 | 0.000 | 9.6E-5 | 0.001 | 0.007 | 0.006 |
| <1.3 | 0.0151204 | 0.000 | 0.000 | 0.000 | 0.000 | 0.000 |
| >0-10 | 0.000 | 0.0151204 | 0.053 | 0.000 | 0.000 | 0.000 |
| <10-20 | 0.000 | 0.000 | 0.015024 | 0.186 | 0.000 | 0.000 |
| <20-30 | 0.000 | 0.000 | 0.000 | 0.0137447 | 0.443 | 0.000 |
| >30 | 0.000 | 0.000 | 0.000 | 0.000 | 0.006 | 0.238 |

| Table 6. Elasticity matrices by period of *Quercus oleoides* population of Mesa de Veinticuatro, Veracruz center state, Mexico. d diameter at breast height (cm), h height (h). | | | | | | |
| --- | --- | --- | --- | --- | --- | --- |
| Cycle 2016-17 | | | | | | |
| Size | Seeds | <1.3 h | >0-10 d | >10-20 d | >20-30 d | >30 d |
| Seeds | 0.000 | 0.000 | 2.9E-4 | 0.001 | 0.002 | 0.020 |
| <1.3 | 0.0231194 | 0.001 | 0.000 | 0.000 | 0.000 | 0.000 |
| >0-10 | 0.000 | 0.0231194 | 0.008 | 0.000 | 0.000 | 0.000 |
| >10-20 | 0.000 | 0.000 | 0.0228239 | 0.036 | 0.000 | 0.000 |
| >20-30 | 0.000 | 0.000 | 0.000 | 0.022216 | 0.071 | 0.000 |
| >30 | 0.000 | 0.000 | 0.000 | 0.000 | 0.020 | 0.750 |
| Cycle 2017-18 | | | | | | |
| Seeds | 0.000 | 0.000 | 1.8E-5 | 0.000 | 0.000 | 0.007 |
| <1.3 | 0.0076422 | 0.001 | 0.000 | 0.000 | 0.000 | 0.000 |
| >0-10 | 0.000 | 0.0076422 | 0.058 | 0.000 | 0.000 | 0.000 |
| >10-20 | 0.000 | 0.000 | 0.0076238 | 0.008 | 0.000 | 0.000 |
| >20-30 | 0.000 | 0.000 | 0.000 | 0.0075868 | 0.020 | 0.000 |
| >30 | 0.000 | 0.000 | 0.000 | 0.000 | 0.007 | 0.869 |
| Cycle 2018-2019 | | | | | | |
| Seeds | 0.000 | 0.000 | 1.8E-4 | 0.001 | 0.006 | 0.010 |
| <1.3 | 0.0177562 | 0.002 | 0.000 | 0.000 | 0.000 | 0.000 |
| >0-10 | 0.000 | 0.0177562 | 0.028 | 0.000 | 0.000 | 0.000 |
| <10-20 | 0.000 | 0.000 | 0.0175731 | 0.077 | 0.000 | 0.000 |
| <20-30 | 0.000 | 0.000 | 0.000 | 0.0166004 | 0.312 | 0.000 |
| >30 | 0.000 | 0.000 | 0.000 | 0.000 | 0.010 | 0.483 |

| Table 7. Elasticity matrices by period of *Quercus oleoides* population of Otates, Veracruz center state, Mexico. d diameter at breast height (cm), h height (h). | | | | | | |
| --- | --- | --- | --- | --- | --- | --- |
| Cycle 2016-17 | | | | | | |
| Size | Seeds | <1.3 h | >0-10 d | >10-20 d | >20-30 d | >30 d |
| Seeds | 0.000 | 0.000 | 0.002 | 0.022 | 0.006 | 0.008 |
| <1.3 | 0.0388227 | 0.073 | 0.000 | 0.000 | 0.000 | 0.000 |
| >0-10 | 0.000 | 0.0388227 | 0.128 | 0.000 | 0.000 | 0.000 |
| >10-20 | 0.000 | 0.000 | 0.0366633 | 0.345 | 0.000 | 0.000 |
| >20-30 | 0.000 | 0.000 | 0.000 | 0.0146926 | 0.058 | 0.000 |
| >30 | 0.000 | 0.000 | 0.000 | 0.000 | 0.008 | 0.219 |
| Cycle 2017-18 | | | | | | |
| Seeds | 0.000 | 0.000 | 0.004 | 0.047 | 0.001 | 0.001 |
| <1.3 | 0.0526897 | 0.022 | 0.000 | 0.000 | 0.000 | 0.000 |
| >0-10 | 0.000 | 0.0526897 | 0.212 | 0.000 | 0.000 | 0.000 |
| >10-20 | 0.000 | 0.000 | 0.0487041 | 0.532 | 0.000 | 0.000 |
| >20-30 | 0.000 | 0.000 | 0.000 | 0.0020077 | 0.000 | 0.000 |
| >30 | 0.000 | 0.000 | 0.000 | 0.000 | 0.001 | 0.025 |
| Cycle 2018-2019 | | | | | | |
| Seeds | 0.000 | 0.000 | 0.003 | 0.037 | 0.003 | 0.003 |
| <1.3 | 0.0458015 | 0.001 | 0.000 | 0.000 | 0.000 | 0.000 |
| >0-10 | 0.000 | 0.0458015 | 0.090 | 0.000 | 0.000 | 0.000 |
| <10-20 | 0.000 | 0.000 | 0.0431863 | 0.583 | 0.000 | 0.000 |
| <20-30 | 0.000 | 0.000 | 0.000 | 0.0064075 | 0.070 | 0.000 |
| >30 | 0.000 | 0.000 | 0.000 | 0.000 | 0.003 | 0.067 |
